# Attitude and perception of pharmacists and healthcare professionals about the criteria used in drug formulary selection in the United Arab Emirates

**DOI:** 10.1186/s40545-022-00460-w

**Published:** 2022-10-18

**Authors:** Sabaa Saleh Al‑Hemyari, Dzul Azri Mohamed Noor, Faris El‑Dahiyat

**Affiliations:** 1 Health and Safety Department, School of Pharmaceutical Sciences, Universiti Sains Malaysia, Pulau Pinang, 11800 Gelugor, Malaysia; 2 Clinical Pharmacy Program, College of Pharmacy, Al Ain University, Al Ain, United Arab Emirates; 3 AAU Health and Biomedical Research Center, Al Ain University, Abu Dhabi, United Arab Emirates

**Keywords:** Pharmacoeconomics, Drug formulary selection, Knowledge, Attitude, Perception, Pharmacist, Healthcare professionals

## Abstract

**Background:**

There is lack of both resources and expertise explains the limited extent to which pharmacoeconomic evidence is used in formulary decision-making.

**Objectives:**

The present study aims to assess attitude and perceptions toward the criteria used to select formulary drugs among UAE healthcare organizations.

**Methods:**

A descriptive cross-sectional study was conducted amongst the licensed physicians in all specialties, all pharmacists and other healthcare professionals with a minimum of 3 month experience those registered with Ministry of health and prevention and those working in the private sector in the UAE. Participants are sent an email containing a validated web-based electronic link to access the questionnaire. The questionnaire composed of two sections is used to assess the healthcare professionals’ attitude and perceptions regarding the criteria used to select formulary drugs. Data analysis were done using SPSS Version 24.

**Results:**

A total of 866 respondents participated in the study and completed the whole questionnaire. The average attitude score about the criteria used in drug formulary selection was 84.5% with a 95% confidence interval (CI) of [83.9%, 85.4%]. Of the total participants, 27 (3.1%) had poor attitude about the criteria used in drug formulary selection, 240 (27.7%) had moderate attitude and 599 (69.2%) had good attitude**.** The results of statistical modeling showed that education level, area of expertise and age were jointly highly associated with attitude about the criteria used in drug formulary selection.

**Conclusions:**

The study revealed that pharmacists and Healthcare professionals had a good attitude about the criteria used in drug formulary selection in the United Arab Emirates. This study purposed to provide Emirate pharmacy and therapeutics policy makers with a clear criterion of best practice related to methodological recommendations to help in increasing the utilization and implementation of pharmacoeconomic evidence in the drug formulary selection process.

## Background

As healthcare expenditure continues to grow, cost currently tops the agenda for stakeholders in this sector. Data gathered in the United States in 2000 indicate that, with 9.4% of all healthcare expenditure allocated to prescription drugs, medications were the primary driver of inflation in the sector [1, 2]. Other western countries have seen similar increases in medicine-related expenditure with studies indicating a real-term rise of over 70% in the period 1990–2001.

Likewise, the nations of the Arabian Gulf have been allocating ever larger proportions of their state budgets to medical and pharmaceutical products [3]. At household level, a 2001 study carried out in Saudi Arabia indicated an average health-related expenditure of $587.50, considerably higher than the mean of $342.50 seen elsewhere in the GCC [4].

In 2018, the United Arab Emirates (UAE) spent a total of $13.7 billion (AED 50.3 billion) on healthcare, including both contributions made to the federal budget by the seven emirates and their own expenditure. Moreover, it was anticipated that this sum would increase by 5.4% to $14.4 billion (AED 53 billion) within a year. Furthermore, Business Monitor International (BMI) has predicted that $26 billion (AED 95.5 billion) will be spent on healthcare, representing 3.6% of the UAE’s GDP, as compared with 3.4% in 2018 [5]**.**

Against this background, considerable pressure has been brought to bear on healthcare providers to ensure cost control and considering safety. A pharmacoeconomic perspective, which entails applying a modified version of health economics methodology with a focus on efficiency, could add value in planning how to maximize benefits received per resource used while also aiding clinicians to factor affordability into their choices [6]. Pharmacoeconomics introduce various strategies to ensure the continued provision of innovative and affordable drugs to gain greater value of money from pharmaceutical expenditure [7]. Decisions in formulary system management must be founded on the clinical, evidence-based, ethical, social, legal, philosophical, logistical, quality-of-life, economic, and safety factors that leads to optimal care of patients [8, 9]. Other different factors shall be considered as well like the degree of the ease of drug use, rates of compliance, taste, dosage forms, and drug stability [10].

Using pharmacoeconomic data is an efficient means to indicate which drugs should be retained or excluded from the formulary and drawing up guidelines for practitioners will enable them to prioritize cost-effectiveness in their medication choices. The formulary is essentially a list of prescription medicines used by a healthcare service, which is subject to regular revisions and updates to ensure it complies with the most recent clinical advice, and which is used by medical staff to diagnose and treat disease [11, 12].

Pharmacy and Therapeutics (P&T) committees are tasked with ensuring drug therapy is safely and effectively delivered [13–15]. The present study aims to assess knowledge and perceptions of, and attitudes toward, the criteria used to select formulary drugs among UAE healthcare organizations. No guidelines have been published to inform decision makers in the UAE about how best to apply pharmacoeconomic evidence. The current study attempts to bridge this gap by providing best-practice methodological guidelines with the ultimate aim of ensuring an increase in the use of pharmacoeconomic evidence in formulary decision-making.

## Methods

### Study design and setting

A descriptive cross-sectional study design was chosen. Accordingly, a survey was circulated to pharmacists and other healthcare professionals registered with the Ministry of Health and Prevention (MOHAP) working in UAE hospitals and pharmacies, as well as their private-sector counterparts, to assess their knowledge and perceptions of, and attitudes toward, the criteria used to select formulary drugs. The survey was conducted over a 1-year period, January 2021–January 2022.

### Target population (inclusion/exclusion criteria)

The study population comprised physicians from all specialties, as well as other MOHAP-registered healthcare professionals and pharmacists, working in public- and private-sector clinics, hospitals, and healthcare centers and with at least 3 month professional experience. The inclusion criteria were employment by MOHAP or any MOHAP-registered healthcare provider, whether public- or private-sector. The exclusion criteria were employment in any non-MOHAP-registered health sector, not being MOHAP-registered, and not having finished a probation period.

### Pilot testing

The survey was pilot-tested by 50 healthcare professionals between January 10, 2021 and February 25, 2021. The data generated by participants in the pilot test were excluded from the final analysis. Twenty-two respondents satisfactorily completed the survey in the pilot test and no difficulties were raised. The findings of the pilot were used to both evaluate the survey’s validity and reliability and to estimate the sample size required for the main survey.

### Sample size calculation

As noted above, the sample size for the final survey was calculated on the basis of the findings of the pilot study. Twenty-two of 50 participants in the pilot returned a completed questionnaire; thus, a response rate of 44% was achieved. We calculated sample size by asking participants if they had ever used pharmacoeconomic data, to which approximately 45% responded that they had. We set the alpha level at 5% to have a 95% confidence interval (CI). The precision (*D*) of the 95% CI is set at 5%, ensuring the width of the latter will be 10% maximum. The assumptions above thus indicated that a sample of *n* = 952 participants was needed with the assumed 60% non-response rate.

### Sampling technique and data collection

The survey was designed to be self-administered. The online survey was performed using the Google Online Survey tool. It was accessed by participants from a web-based electronic link emailed to them after the email addresses of every registered physician, pharmacist, and healthcare professional in the UAE were obtained from MOHAP. In the current study, MOHAP provide the sampling frame including the healthcare professional’ email address, position and department. This would minimize selection bias, guarantee a high response rate, avoid he inconvenience, and improve the generalizability of the study. The cover page contained information about the purpose of the research. The participants were first asked to indicate their desire to participate in the survey. All participants who proceeded to the next page were understood to have given informed consent. Respondents who did not complete the survey were emailed monthly reminders, and those who did complete it were thanked. No financial or other incentive was offered to participants.

### Research instrument development

The researchers reviewed the relevant literature on knowledge and perceptions of, and attitudes toward, formulary management and pharmacoeconomics [15–17], after which they created a structured questionnaire which was suitable for self-administration. The researchers then drew on similar instruments found in the literature to adapt the questionnaire to a UAE context and ensure that it addressed the salient points of the research question of this study. Thereafter, it was sent for expert review by seven professionals in the fields of pharmacoeconomics and clinical pharmacy at the Universities of Ajman, Al Ain, and USM, who were asked to ensure the relevance and appropriateness of its content. After the experts had validated the instrument and given feedback, the final minor modifications were made.

To assess the content validity ratio and content validity index (CVR/CVI) of the survey used, experts were asked to rate all items as essential or non-essential. A CVR equal to or exceeding 0.78 is considered good [18]. Normally, individual items which do not reach this threshold are eliminated from the survey, after which it is possible to calculate CVI from a mean of all CVR values per item in the final version, that is, all those items which achieve a CVR equal to or greater than 0.78. In our case, the final version of instrument achieved an acceptable CVI, namely, 0.878 [19]**.** Thereafter, 50 healthcare experts piloted the survey to evaluate its face validity; their data were excluded from the final analysis. Cronbach’s α was used to rate the questionnaire’s reliability. The calculated coefficient was 0.76, indicating that its internal consistency was acceptable.

### Research instrument sections

The questionnaire consists of two sections. It is designed to evaluate knowledge and perceptions of, and attitudes toward, the criteria used to select formulary drugs among pharmacists and healthcare professionals.

1. Questions in the first section aimed to gather demographic data, such as age, gender, level of education, years of experience, health organization/affiliation, area of expertise, and whether the individual had ever used pharmacoeconomic data.
2. The 21 questions contained in the second section addressed the criteria used in selecting formulary drugs in UAE health organizations. Participants were asked to rate the degree of importance of certain strategies in influencing the process of selecting formulary drugs in the UAE as (1) not influential, (2) slightly influential, (3) moderately influential, (4) influential, or (5) very influential.

### Questionnaire scoring

21 items assessing participants’ attitude and perceptions toward, the criteria used to select formulary drugs were assessed by a 5-point Likert scale (where 1 = “not influential”, 2 = “slightly influential”, 3 = “moderately influential”, 4 = “influential,” and 5 = “very influential”). Thereafter, the overall grade of each item was calculated by summing the raw Likert-scale scores. The percentage (0–100%) calculated for each participant thus indicated their general attitudes toward, the criteria used to select formulary drugs.

The original Bloom's cutoff points were updated and adjusted to evaluate UAE pharmacists' and healthcare professionals' general attitude toward criteria employed in the Drug Formulary selection process. [20–24]

The overall attitude was classified as good if the score was between 80% and 100%, moderate if the score was between 60% and 79%, and poor if the score was less than 60%, according to Bloom's cutoff point. Accordingly, the overall attitude score for the criteria employed in the Drug Formulary selection process was classed as good for a score between 84 and 105 points, moderate for a score between 63 and 83 points, and poor for a score of less than 63 points, using the same Bloom's cutoff point.

### Ethical considerations

The Ethical Review Committee of MOHP approved this study. The study’s purpose was stated on the cover page of the questionnaire, and all participation was voluntary. Participants were deemed to have given their consent by proceeding to the second page. No records were kept of participants’ identity, and confidentiality was maintained throughout the study process.

### Statistical analysis

SPSS Version 24 was used to analyze the data generated. Frequencies (as percentages) were used to summarize the qualitative variables and ± standard deviation (± SD) to summarize the quantitative ones. As regards the quantitative variables, unpaired Student *t* tests, non-parametric versions, and one-way ANOVAs were also used to measure cross-group differences. Assessment for normality was carried out using Shapiro–Wilk test (with *p* > 0.05 indicating a normally distributed continuous variable) or by visual inspection of a Normal *Q*–*Q* Plot Logistic regression models were used to identify factors impacting the attitude and perceptions toward, the criteria used in selecting formulary drugs process among healthcare providers. The stepwise method was used for variable selection and model building. A *p* value of < 0.05 was set to indicate statistical significance.

## Results

### Demographic information of the study participants’

Table 1 presents the demographic information. A total of 866 respondents participated in the study and completed the whole questionnaire. The average age of respondents was 42.1 ± 8.8 SD. Of the total participants 27.7% (*n* = 240) were male and 72.3% (*n* = 626) were female. Among the participants, 70.6% (*n* = 611) were Bachelor degree holders, 19.6% (*n* = 170) were master degree holders, 4.8% (*n* = 42) have Pharm D education and 5% (*n* = 43) were PhD holders. About three quarters (*n* = 637, or 73.6%) were from hospitals and 26.4% (*n* = 229) from primary healthcare centers. The years of experience among the participants were as follows: 61 (7%) had 1 to 5 year experience, 176 (20.3%) had 6–10 year experience, 355 (41%) had 11–20 year experience and 274 (31.6%) had more than 20 year experience. Nearly, third of the participants (35.6%, 308) had ever used Pharmacoeconomic data. The area of expertise amongst the participants was detailed as follows: 497 (57.4%) from Nursing, 14 (1.6%) were Regulatory Staff, 25 (2.9%) from Community Pharmacy, 124 (14.3%) from clinical pharmacy, 124 (2.3%) from Dentistry, 174 (20.1%) were Physicians and 12 (1.4%) were other paramedical members.

**Table 1:** Number and percentage of the questions on demographic information (*n* = 866)

Demographics	Groups	Frequency	Percentage (%)
Gender	Male	240	27.7
	Female	626	72.3
Education	Bachelor	611	70.6
	Master's degree	170	19.6
	Pharm D	42	4.8
	PhD	43	5
Health organization	Hospital	637	73.6
	PHC	229	26.4
Experience years	1–5 years	61	7
	6–10 years	176	20.3
	11–20 years	355	41
	more than 20 years	274	31.6
Area of expertise	Nursing	497	57.4
	Regulatory Staff	14	1.6
	Community Pharmacy	25	2.9
	Hospital pharmacy/clinical pharmacy	124	14.3
	Dentistry	20	2.3
	Other paramedical members	12	1.4
	Physician	174	20.1
Ever use pharmacoeconomic data	Yes	308	35.6
	No	558	64.4

*PHC* Primary Health care centers

### Assessment of attitude about the criteria used in drug formulary selection

The average attitude score about the criteria used in drug formulary selection was 84.5% with a 95% confidence interval (CI) of [83.9%, 85.4%]. Of the total participants, 27 (3.1%) had poor attitude about the criteria used in drug formulary selection, 240 (27.7%) had moderate attitude and 599 (69.2%) had good attitude**.** The attitude about the criteria used in drug formulary selection was evaluated by 21 items. Contents of sodium chloride, sugars, and lactose as well as number of (un) registered indications and Potential of patient/staff abuse of the drug were the lowest criteria chosen by the participants (Table 2).

**Table 2:** Assessment of attitude about the criteria used drug formulary selection

Criteria used in drug formulary selection	Mean	± SD	Median	influential/very influential	
				*F*	(%)
1. Number, frequency and severity of adverse drug reactions	4.31	0.86	5	696	80.4
2. Safety or frequency and severity of toxicity	4.37	0.83	5	712	82.2
3. Antibiotic resistance	4.35	0.84	5	706	81.5
4. Clinical evidence-based effectiveness in scientific literature	4.27	0.84	4	694	80.1
5. Effect of the drug on the quality of life	4.33	0.84	5	713	82.3
6. Medical specialists clinical expertise	4.23	0.86	4	687	79.3
7. Use in children or neonates	4.21	0.95	5	660	76.2
8. Number and severity of contraindications	4.29	0.86	5	696	80.4
9. Specific characteristics of hospital patient population	4.12	0.92	4	645	74.5
10. Drug–drug interaction	4.29	0.88	5	686	79.2
11. Therapeutic window	4.21	0.86	4	672	77.6
12. Use during childish, pregnancy, and lactation	4.28	0.88	5	687	79.3
13. Drug–food interactions	4.24	0.88	4	679	78.4
14. New and innovative pharmacological effect	4.17	0.89	4	663	76.6
15. Number of (un)registered indications	4.07	0.93	4	629	72.6
16. Contents of sodium chloride, sugars, and lactose	4.01	0.98	4	605	69.9
17. Selected drug cost	4.16	0.87	4	659	76.1
18. Presence of alternatives in the current formulary	4.17	0.88	4	655	75.6
19. Potential of patient/staff abuse of the drug	4.13	0.94	4	640	73.9
20. Evidence-based practice ( approved treatment protocols)	4.29	0.85	5	688	79.4
21.Patients convenience/patients adherence	4.23	0.86	4	673	77.7

*F* frequency, % Percentage, *SD* standard deviation

Table 3 shows the distribution of the attitude score about the criteria used in drug formulary selection according to demographic information. The table provides also the 95% confidence intervals for the estimates and the *p* values. These *p* values were computed from the findings of the independent sample *t* test and one-way ANOVA.

**Table 3:** Participants’ attitude about the criteria used in drug formulary selection according to demographics

Demographics	Groups	*N*	Mean	± SD	95% CI		*p* value
					Lower	Upper	
Gender	Male	240	90.51	12.98	88.86	92.16	0.032*
	Female	626	88.05	15.84	86.80	89.29	
Education	Bachelor	611	89.24	15.67	87.99	90.48	0.121
	Master's degree	170	86.34	14.58	84.13	88.55	
	Pharm D	42	88.80	11.68	85.16	92.45	
	PhD	43	90.93	11.51	87.38	94.47	
Health organization	Hospital	637	88.30	14.70	87.16	89.45	0.167
	PHC	229	89.92	16.28	87.80	92.04	
Experience years	1–5 years	61	87.96	15.47	84.00	91.92	0.392
	6–10 years	176	88.21	15.07	85.97	90.45	
	11–20 years	355	88.11	15.75	86.47	89.75	
	> 20 years	274	90.04	14.28	88.34	91.74	
Area of expertise	Nursing	497	88.58	15.65	87.20	89.96	0.018*
	Regulatory Staff	14	77.21	26.66	61.81	92.61	
	Community Pharmacy	25	93.40	11.83	88.51	98.28	
	Clinical pharmacy	124	89.80	13.84	87.34	92.26	
	Dentistry	20	91.30	13.68	84.89	97.70	
	Other paramedical members	12	96.16	9.40	90.18	102.14	
	Physician	174	87.83	13.72	85.78	89.89	
Ever use pharmacoeconomic data	Yes	308	90.01	14.37	88.40	91.62	0.065
	No	558	88.03	15.52	86.73	89.32	

*PHC* Primary Health care centers, *SD* standard deviation, *CI* confidence interval

*: (*P* ≤ 0.05) is statistically significant

The male participants scored better in the attitude about the criteria used in in drug formulary selection compared to female participants (*P* = 0.032). The average attitude score was 90.51 for males and 88.05 for females.

There was a statistically significant difference in the attitude about the criteria used in drug formulary selection according to area of Expertise (*P* = 0.018). The results of Tukey post hoc test showed that other paramedical members scored better in the attitude about the criteria used in Pharmacoeconomic methodology evaluation compared to regulatory staff (*P* = 0.024) and compared to community pharmacists (*P* = 0.023).

### Factors influencing the attitude about the criteria used in drug formulary selection

Table 4 displays the results of univariate and multivariate logistic regression models to assess the influence of demographic variables on the healthcare professionals' attitude about the criteria used in drug formulary selection. From the univariate analysis, better attitude about the criteria used in drug formulary selection was observed in older participants (OR 1.031; 95% CI 1.014–1.049). However, poor attitude about the criteria used in drug formulary selection was observed in master degree holders (OR 0.542; 95% CI 0.381–0.770) and regulatory staff (OR 0.238; 95% CI 0.078–0.722).

**Table 4:** Univariate and multivariate regression analysis for the factors influencing the participants’ attitude about the criteria used in drug formulary section

Factors	Good attitude about the criteria used in drug formulary selection (≥ 84)							
	Univariate				Multivariate			
	OR	95% CI		*p* value	OR	95% CI		*p* value
Gender (Ref. Female)								
Male	1.212	0.873	1.683	0.25	–	–	–	–
Education (Ref. Bachelor)								
Master's degree	0.542	0.381	0.770	0.001*	0.528	0.348	0.801	0.003*
Pharm D	0.995	0.498	1.989	0.990	–	–	–	–
PhD	1.504	0.707	3.202	0.289	–	–	–	–
Health organization (Ref. PHC)								
Hospital	0.829	0.594	1.157	0.271	–	–	–	–
Experience years (Ref. 1–5 years)								
6–10 years	0.943	0.508	1.751	0.853	–	–	–	–
11–20 years	1.018	0.571	1.816	0.952	–	–	–	–
> 20 years	1.369	0.752	2.490	0.304	–	–	–	–
Ever use pharmacoeconomic data (Ref. No)								
Yes	1.125	0.831	1.524	0.446	–	–	–	–
Area of expertise (Ref. Nursing)								
Other paramedical members	7.612	0.871	33.781	0.713	–	–	–	–
Regulatory staff	0.238	0.078	0.722	0.011*	0.228	0.072	0.726	0.012*
Community pharmacy	1.713	0.631	4.649	0.291	–	–	–	–
Clinical pharmacy	0.933	0.610	1.427	0.750	–	–	–	–
Dentistry	0.999	0.377	2.650	0.998	–	–	–	–
Physician	0.835	0.578	1.206	0.335	–	–	–	–
Age	1.031	1.014	1.049	< 0.001*	1.031	1.013	1.050	0.001*

Forward and backward stepwise procedure was used in multiple logistic regression model

*PHC* Primary Health care centers, *OR* odds ratio, *CI* confidence interval

*: (*P* ≤ 0.05) is statistically significant

To determine the set of factors that jointly influences the attitude about the criteria used in drug formulary selection, a stepwise procedure in a multiple logistic regression model was used. The results of this procedure showed that education level, area of expertise and age were jointly highly associated with attitude about the criteria used in drug formulary selection.

## Discussion

The current study aimed to evaluate knowledge and perceptions of, and attitudes toward, the criteria used to select formulary drugs among UAE healthcare organizations. Typically, drugs are selected depending on specific criteria, including cost, quality, safety, and efficacy. The formulary is also required to be consistent with any regional or national formulary treatment guidelines. The study found an average attitude score of 84.5%, indicating that several people had a good attitude about the criteria used in drug formulary selection. In detail, 69.2% had a good attitude, 27.7% had a moderate attitude, and only 3.1% had a poor attitude. A substantial percentage of the participants indicated that they have a good attitude indicating they are optimistic about the criteria used to select formulary drugs. A study by Matiala et al. reveal that there is difference in the medication review process based on costs, patient safety, and clinical experience [25]. Furthermore, Sharma et al. did a similar study in India and found that many practitioners believe that a well-developed formulary can advance the public health care system's quality [26]. Moreover, the study indicates that the practitioners noted that using formulary medicines reduces their autonomy and restricts the flexibility and individuality of the patient [26]. The study also found that Males have a better attitude about the criteria used in drug formulary selection when compared to their female counterparts. Again, Alarifi [27] and Bilal et al. [28] report that most healthcare professionals have knowledge and positive attitude toward generic medicine and its selection criteria. Bagga [29] also reports that pharmacists and doctors are knowledgeable about the use of formulary. Furthermore, Yimenu et al. [30] and Alsuhebany et al. [31], reveal that most doctors and pharmacists prefer know about and prefer the criteria deployed in drug formulary selection. The International Pharmaceutical Federation contend that drug formulary selection criteria is part of good pharmacy practice [32]. Besides, Seid et al. [33] and Goshime [34] also reveal that pharmacists have a more knowledge about drug formulary selection criteria and adverse drug reaction than other healthcare professionals.

In the study, participants' areas of expertise made a significant statistical difference in the attitude about the criteria used in drug formulary selection. Compared to community pharmacists and regulatory staff, paramedical members were found to have a better attitude about the criteria used in drug formulary selection. Older participants were also found to have a better attitude about the criteria used in drug formulary selection than younger participants.

Moreover, it was noted that a significant number of participants who had master's degrees and those in the regulatory staff area of expertise had a substantially poor attitude about the criteria used in drug formulary selection. In Brazil, Alcântara et al. reveal that pharmacists have poor attitude toward drug formulary selection process due to inadequate knowledge and education [35]. Again, Hayat found that healthcare professionals perceive pharmacists as the source of information, meaning that pharmacists are more knowledgeable about the drug formulary selection criteria [36]. The study depicts that the master's degree participants and regulatory staff had some different knowledge that made them have a poor attitude about the criteria. In general, the study results depict that age, area of expertise, and education level are jointly highly linked with the attitude about the criteria used in drug formulary selection. On the other hand, in UAE, effective strategies to manage medications in the formulary include the use of generic medications, use of biosimilars to minimize the cost of using biologics, guided-use policies, therapeutic interchange, evidence-based clinical guidelines, antibiotic stewardship programs, using the smart pharmacoeconomic tools in the computerized formulary system with a quick reference guide available to ease the use of the system, awareness programs on formulary management, and Medication Use Evaluation.

## Conclusions

The study revealed that pharmacists and Healthcare professionals had a good attitude about the criteria used in drug formulary selection in the United Arab Emirates. Females and younger people were found to have a poorer attitude toward the criteria than males and older people. Another significant result of the study is that several regulatory staff and master's degree participants were found to have a poor attitude toward the criteria. A formulary system is an evidence-based multidisciplinary process employed by organizations to add and use drugs that has the best therapeutic effect while decreasing potential costs and risks for patients. Professionals who are involved in the drug-use process have to know how the organization’s policies and processes shall be integrated into their daily tasks to ensure the drugs are used safely and appropriately. Technology offers several opportunities to enhance the efficiency of those processes. Communicating the actions pertaining to drugs use is a constant burden that organizations have to address. Pharmacoeconomic is a crucial aspect that can promote cost control in healthcare organizations. Application of formulary management and pharmacoeconomic can reduce the significant rise in healthcare expenses by identifying ways to manage the raised costs. The goal is to promote efficiency by increasing the benefits received per resource used. The study suggests that lowering drug expenditure and the general healthcare costs involves proper controlling of formularies. It’s advisable to develop a tool consisting of a different domain checklist of questions to evaluate medications in the request queue.

The tool shall pose different questions related to: need evidence, medication safety, efficacy, potential of misuse, Pharmacoeconomics, and the decision-making process.

This is to facilitate a more standardized and effective decision making. In addition, this approach is capable of educating fresh committee members, facilitate discussions of medications requested for the formulary addition, and can even be used to assess the quality of the committee decision making.

## Data Availability

The data that support the findings of this study are available from the corresponding author upon reasonable request.
